# Role of Oceanic and Terrestrial Atmospheric Moisture Sources in Intraseasonal Variability of Indian Summer Monsoon Rainfall

**DOI:** 10.1038/s41598-017-13115-7

**Published:** 2017-10-06

**Authors:** Amey Pathak, Subimal Ghosh, Praveen Kumar, Raghu Murtugudde

**Affiliations:** 1 Department of Civil Engineering, Indian Institute of Technology Bombay, Mumbai, 400 076 India; 2 Interdisciplinary Program in Climate Studies, Indian Institute of Technology Bombay, Mumbai, 400 076 India; 3 Civil and Environmental Engineering, University of Illinois at Urbana–Champaign, 2527B VenTe Chow Hydrosystems Laboratory, 301N. Mathews Ave., 61801 Urbana, IL USA; 4 Earth System Science Interdisciplinary Center (ESSIC)/DOAS, University of Maryland, College Park, Maryland USA

## Abstract

Summer Monsoon Rainfall over the Indian subcontinent displays a prominent variability at intraseasonal timescales with 10–60 day periods of high and low rainfall, known as active and break periods, respectively. Here, we study moisture transport from the oceanic and terrestrial sources to the Indian landmass at intraseasonal timescales using a dynamic recycling model, based on a Lagrangian trajectory approach applied to the ECMWF–ERA–interim reanalysis data. Intraseasonal variation of monsoon rainfall is associated with both a north-south pattern from the Indian landmass to the Indian Ocean and an east-west pattern from the Core Monsoon Zone (CMZ) to eastern India. We find that the oceanic sources of moisture, namely western and central Indian Oceans (WIO and CIO) contribute to the former, while the major terrestrial source, Ganga basin (GB) contributes to the latter. The formation of the monsoon trough over Indo-Gangetic plain during the active periods results in a high moisture transport from the Bay of Bengal and GB into the CMZ in addition to the existing southwesterly jet from WIO and CIO. Our results indicate the need for the correct representation of both oceanic and terrestrial sources of moisture in models for simulating the intraseasonal variability of the monsoon.

## Introduction

Understanding the variability of Summer Monsoon Rainfall in Indian sub-continent is of utmost importance as it affects the food and water security of more than a billion people. The Indian Summer Monsoon (ISM)is generally spread across June-September and contributes to 70–80% of annual rainfall in India. The Indian Summer Monsoon Rainfall (ISMR) is associated with variabilities at different timescales^1–3^, viz., intraseasonal, interannual and multi-decadal and they are reported to be inter-related^4–6^. Large scale and regional scale processes substantially contribute to these wide ranges of rainfall variabilities and understanding of these processes is crucial for reliable monsoon predictions at sub-seasonal to seasonal scales and projections at decadal to century scale.

ISMR is governed by a strong cross-equatorial moisture transport^7^ through the western Indian Ocean and the recycling^8^ of moisture generated from Indian landmass. The intraseasonal variability in the ISM is associated with the fluctuations of the Tropical Convergence Zone (TCZ)^1,9^. This is generally observed in the form of active and break periods associated with high and low rainfall anomalies, respectively, over the core monsoon zone (CMZ)of central India. Breaks during summer monsoon are often associated with migration of monsoon trough^10^ to the Himalayan foothills, anticyclonic vorticity^10^ at 850 hPa, and a southward protrusion of mid-latitude trough into northern India^11,12^. Active period are associated with formation of low pressure systems (LPS)^10^, increased convection^2,13^, moisture convergence^14–16^ over CMZ, a strong cyclonic vortex over the north Bay of Bengal^2,10^, a strengthening of Hadley circulation over the monsoon domain^13^ and a strengthening of east–west Walker circulation over the Pacific. It is believed that the occurrence of LPS over the monsoon region is nearly 3.5 times higher^16^ duringactive periods than those during a break. Furthermore, the intraseasonal variability of active and break cycles is primarily associated with westward (a quasi bi–weekly mode; 10–20 days)^13,14,17–21^ and northward (30–60 days)^9,13,20,22^ propagating convection and circulation features. An understanding of the origin of these two dominant modes of intraseasonal variability, viz., 10–20 days and 30–60 days are stillevolving^13^ and their contribution to the seasonal mean rainfall is also debated^13^. We resort to a composite analysis of active and break cycles to capture the most robust features in moisture source variability. Prolonged or frequent break periods during the monsoon season may lead to drought conditions^2,17,23^. Such a break period during the critical growth stage of agricultural crops lead to a substantially reduced yield^23^. A recent study^24^ reports an increase in the frequency of dry spells with a decrease in rainfall during break periods but an increase in intensity of active periods over the last 50 years. This further underscores the need for further understanding the active and break cycles and prediction of their characteristics for adaptation of agriculture and water management to climate variability and change^25^, especially considering the inadequacies of the current climate models^26,27^. The lack of skill is partly attributable to inadequate understanding of synoptic scale processes such as the Monsoon Intra Seasonal Oscillation **(**MISO)^27,28^, Madden Julian Oscillation (MJO)^29–32^, migration of the Inter-Tropical Convergence Zone (ITCZ)^1,9^ to the northern latitudes, convection over the Bay of Bengal^33^, interactions of ISMR with aerosols through direct radiative forcing^34^, and the atmospheric moisture transport^35^ to the Indian subcontinent. Understanding of the moisture source availability and its variability will advance our understanding of all these related processes. This will also help us understand the performance of state of the art climate models in simulating the intra-seasonal variability of Indian monsoon and the associated processes.

**Figure 1:**
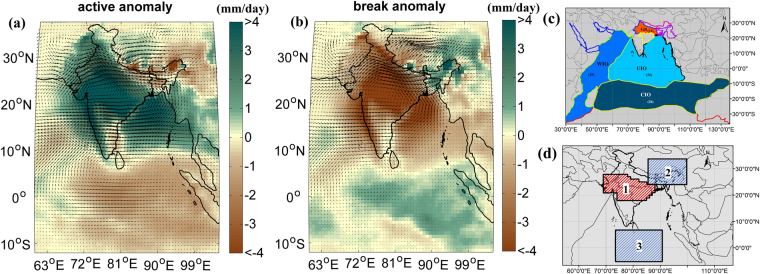
Precipitation anomalies during (**a**) active and (**b**) break periods in the Indian Summer Monsoon. An active period is defined as the period of 3–15 days, during which the rainfall anomaly over the core monsoon zone (region 1 in (**d**)) is positive. Similarly a break period denotes the period of negative rainfall anomaly over region 1. Major sources of moisture to ISMR are shown in (**c**). East–west asymmetry in precipitation anomaly is observed between the core monsoon zone [region 1] and region 2. Similarly a north–south asymmetry in precipitation anomaly is observed between regions 1 and 3, during active (**a**) and break periods (**b**). The color scale runs from <−4 to >4 in intervals of 1 *mm day* ^*−*1^. The anomaly values are statistically significant at 0.05 level. 2-tailed student’s t-test is used for the same. Maps are prepared using MATLAB R2012b (http://in.mathworks.com/products/new_products/release2012b.html) and ESRI 2011-ArcGIS Desktop: Release 10. Redlands, CA: Environmental Systems Research Institute. The terrestrial and oceanic boundaries used in the plot are developed from the free spatial data provided by DIVA-GIS website (http://www.diva-gis.org/), Pathak *et al*.^37^ and MATLAB R2012b.

The major evaporative sources that contribute to the intraseasonal variability of ISMR are not fullyunderstoodyet^36^. As the large-scale disturbances primarily originate over the oceans and terrestrial mid–latitudes regions, the understanding of the monsoon intraseasonal variations can be further improved by studying the atmospheric moisture transport from oceanic and terrestrial sources that ultimately determine the precipitation totals and their distribution over Indian subcontinent. Furthermore, at present there are relatively few systematic studies that address the issues of rainfall variability that arise due to the atmospheric moisture availability and its transport^37^ leaving open the questions on how and to what extent the variabilities in moisture transport from different oceanic and terrestrial sources contribute to intraseasonal timescales. The intra-seasonal variability of ISMR is defined as the rainfall anomaly over the Core Monsoon Zone (CMZ). CMZ^15^ extends roughly from 18°N to 27°N and 69°E to 88°E [Fig. 1(d), Region1]. The monsoon rainfall over the CMZ is significantly correlated with the rainfall over major parts of India^15^. Here, we analyze the transport of moisture from different evaporative sources to the CMZ^15^ during active and break periods. The analysis is performed using a modified dynamic recycling model based on a Lagrangian trajectory approach^38,39^ at daily timescale with a spatial resolution of 0.75° × 0.75°. The percentage contribution from and variability of different moisture sources contributing to intraseasonal oscillation of ISMR are quantified.

## Results and Discussions

Active and break periods of ISMR are identified^15^ using daily precipitation data from ERA–Interim^40^ (European Centre for Medium–Range Weather Forecasts Reanalysis; details are presented in Method section). The spatial pattern and distribution of precipitation from ERA–interim during active and break periods closely resemble the gridded precipitation data of India Meteorological Department^41^ [Supplementary Figure S1]. A monsoon trough^10^ builds up initially over northwest India and then extends down to the Bay of Bengal through the CMZ [Fig. 1(d)] via a strong low pressure system^10^, which delivers high precipitable water and rainfall over the CMZ during active periods (Fig. 1). In contrast, during a break period, high rainfall occurs over northeast India^11,12^ (Fig. 1) and equatorial Indian Ocean. The high rainfall over northeast India is due to the migration of this low pressure system to the Himalayan foothills resulting low rainfall over the CMZ, simultaneously [Fig. 1(b)]. The formation of the monsoon trough is also associated with the TCZ migration^1^ whilethe resulting fluctuations between the periods of high and low rainfall within the summer monsoon season essentially create a competition for rainfall between the Indian subcontinent and the tropical Indian Ocean^1^. A northward shift of the equatorial TCZ is observed during active periods while the TCZ is anchored over the ocean during break periods [Supplementary Figure S2(a,b)]. This TCZ meridional migration is largely responsible for the north–south contrast in rainfall [Fig. 1(a and b)] between the CMZ and equatorial Indian Ocean [*region* 1 and *region 3* in Fig. 1(d)]with its phase essentially reversing during active and break periods. The intraseasonal variation in the Indian summer monsoon is associated with the competition for moisture convergence among the three regions, i.e., the CMZ (region1), northeast India (region2) and the equatorial Indian Ocean (region3) [Fig. 1(d)].To investigate this interesting feature of ISMR, we study the atmospheric moisture transport from major evaporative sources [Fig. 1(c) and Supplementary Figure S3(b)] to the Indian monsoon region during active and break periods. Among the oceanic sources, the Western Indian Ocean (WIO), Central Indian Ocean (CIO), and Upper Indian Ocean (UIO) are the major contributors of atmospheric moisture loading; whereas Ganga basin (GB) is the major source among the land evaporative sources [Supplementary Figure S3(b)]. The rationale behind the selection these regions may be found in Pathak *et al*.^37^.

**Figure 2:**
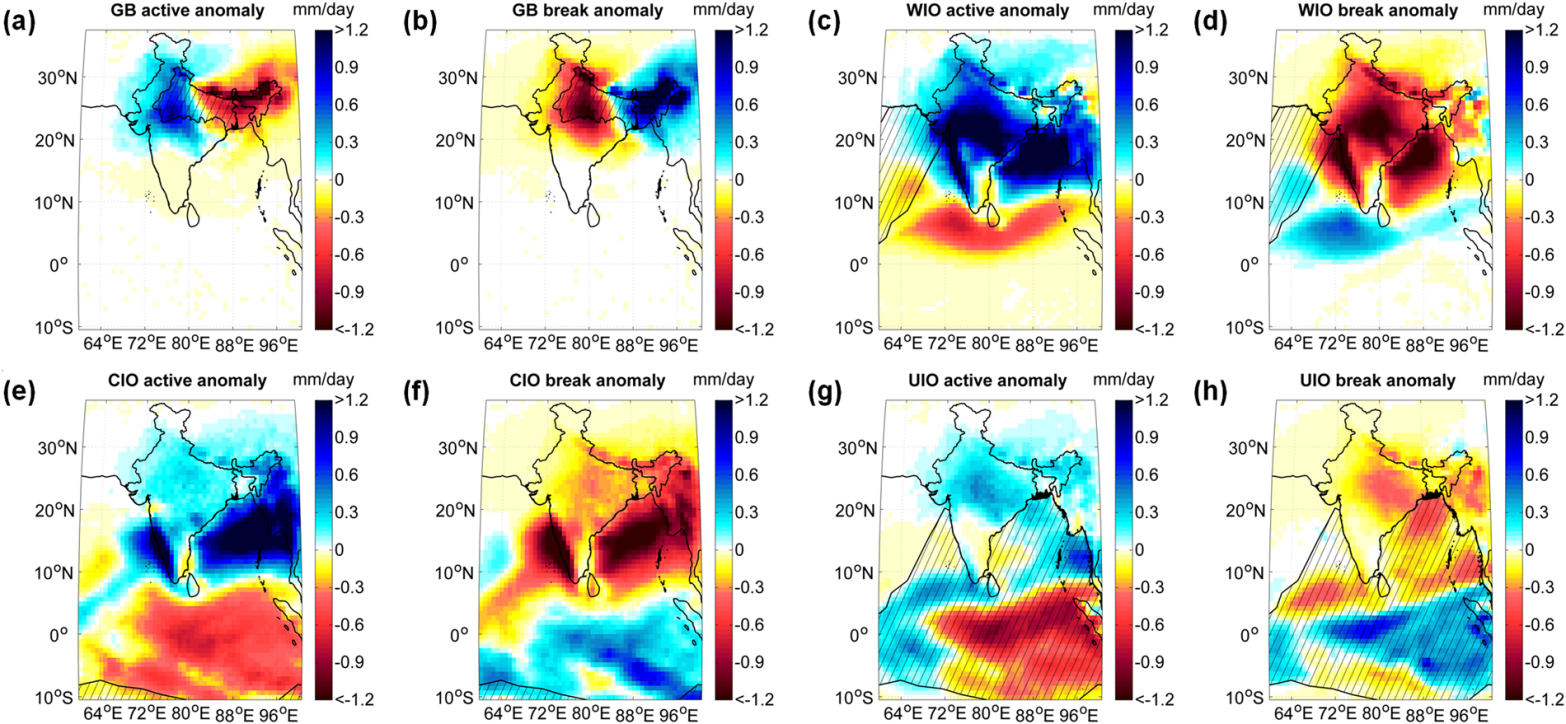
Anomalies of the moisture contributions from the major sources during active and break periods. During active and break periods, east–west asymmetry is observed in the contributions from the terrestrial source, i.e., the Ganga Basin (GB), whereas the north–south asymmetry is observed in the contributions from the oceanic sources (central Indian Ocean (CIO), western Indian Ocean (WIO), and upper Indian Ocean (UIO)). Here, hatched areas denote the source regions. It is important to note that the total spatial extent of the source regions are shown in Supplementary Figure S3(a). Here, all units are in *mm day* ^*−*1^. The anomaly values are statistically significant at 0.05 level. 2-tailed student’s t-test is used for the same. Maps are prepared using MATLAB R2012b (http://in.mathworks.com/products/new_products/release2012b.html). The terrestrial boundaries used in the plot are developed from Pathak *et al*.^37^, free spatial data provided by DIVA-GIS website (http://www.diva-gis.org/), and MATLAB R2012b.

Bulk of the moisture supply to the ISMR is traditionally assumed to be transported by the Somali Low Level Jet (SLLJ). While there is a strong association between Somali Low Level Jet and ISMR^42^, the intraseasonal variability of ISMR is not fully explained by that of Somali Low Level Jet. The WIO is indeed a major evaporative source^36^ contributing to ISMR although contributions from the CIO, UIO and GB^37^ are also important. It is thus important to understand their individual roles in intraseasonal variability of ISMR which has not been addressed in the literature. The role of land surface evapotranspiration^8^ during active/break periods also needs to be understood. Furthermore, intraseasonal variation is viewed as a predominant north-south pattern^1,9^ in precipitation but the clearly evident east–west^12^ variability has not received much attention. We shed some light on these issues here with the help of moisture transport from different major sources. Figure 2 shows anomalous contribution of moisture to precipitation (calculated with eq. 2) over the Indian monsoon region from different moisture sources during active and break periods. An east–west phasing is observed in the moisture contributions from the terrestrial source of GB, whereas north–south pattern is associated with the moisture contributions from the oceanic sources represented by CIO, WIO, and UIO. The mechanisms behind these east–west and north–south rainfall patterns are explored in detail in following sub-sections:

### East–West pattern of rainfall during Active and Break periods

Break conditions during the summer monsoon are generally associated with heavy rainfall over the Himalayan foothills and northeast India [Fig. 1(b)]. During the break periods, the monsoon trough shifts to the Himalayan foothills and the pressure over the rest of India tends to be above normal. This northward migration of the trough is associated with an interruption of monsoonal easterlies by strong subtropical westerlies resulting from a southward protruding mid-latitude trough^11,12^. A prominent northwesterly circulation [Fig. 1(b)] develops over the CMZ during the break period resulting in an advection of terrestrial moisture from the Ganga Basin (GB) to northeast India. An enhancement of precipitation over the Himalayan foothills is also partly associated with the increased atmospheric instability due to the orographiclift^12^. In contrast to this monsoon dance at the Himalayan foothills, during active conditions, the southwesterly jet becomes prominent [Fig. 1(a)] and a strong monsoon trough forms over the CMZ which delivers copious amounts of rain to the CMZ and most of India except the northeast and the southeastern peninsular region [Fig. 1(a)]. The moisture evaporated from GB precipitates over the core monsoon region during an active period whereas it is transported to the northeast during a break period [Fig. 2(a,b)]. We observe that the high recycling within the GB is an important feature during active periods [Fig. 2(a)].

**Figure 3:**
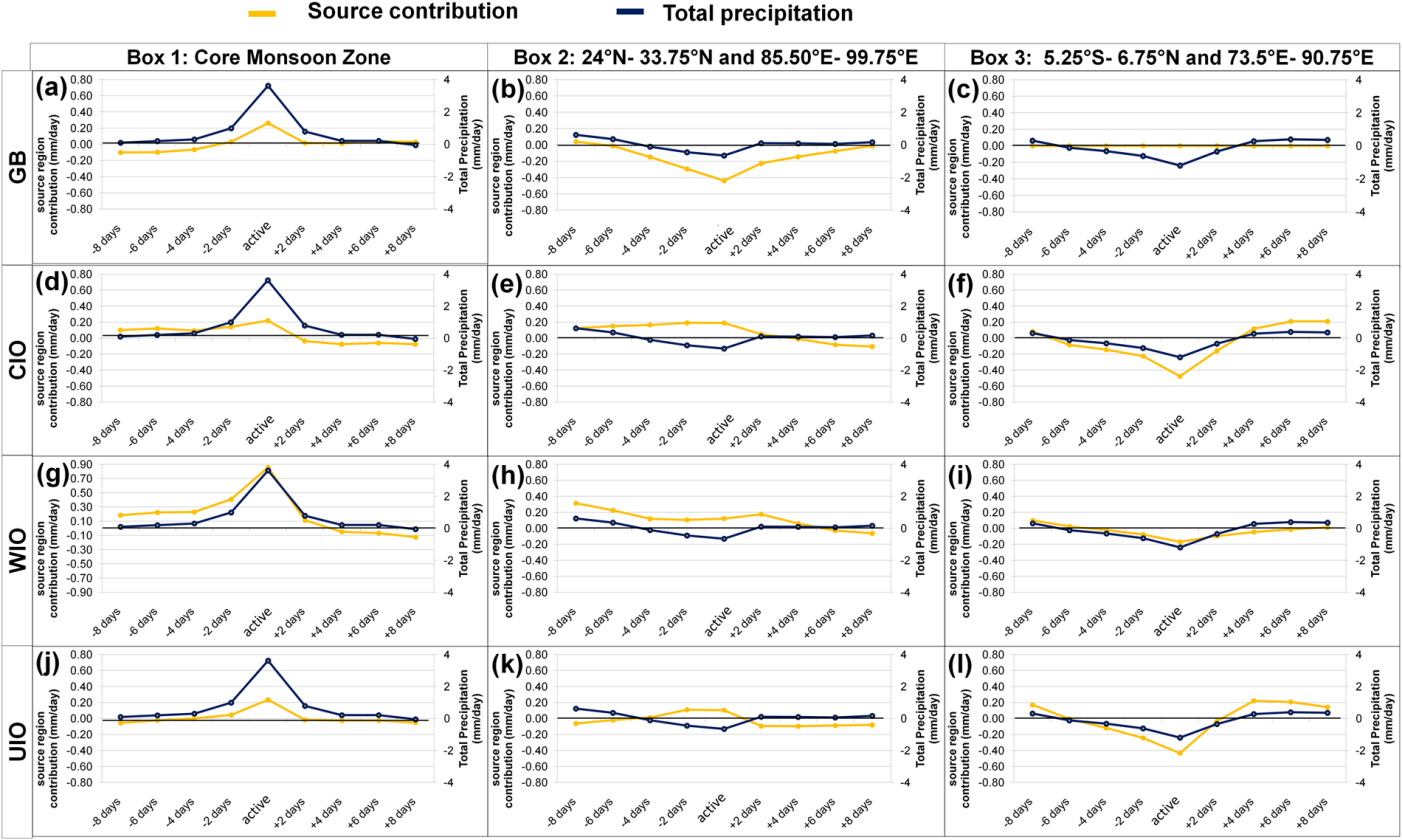
Anomalies of moisture contribution from the major sources to precipitation over the three regions (**c**), before, during and after an active period. The three regions as shown in (**c**) are the same as that in Fig. 1(d). Here, the yellow and blue lines represent the anomalies of moisture contribution from the moisture source region and the total precipitation respectively with their corresponding markings on left-hand side and right-hand side of vertical scale, respectively. The vertical-axis units are in *mm day* ^*−*1^. Plots are prepared using Microsoft Excel 2007 (https://products.office.com/en-IN/download-office-2007).

**Figure 4:**
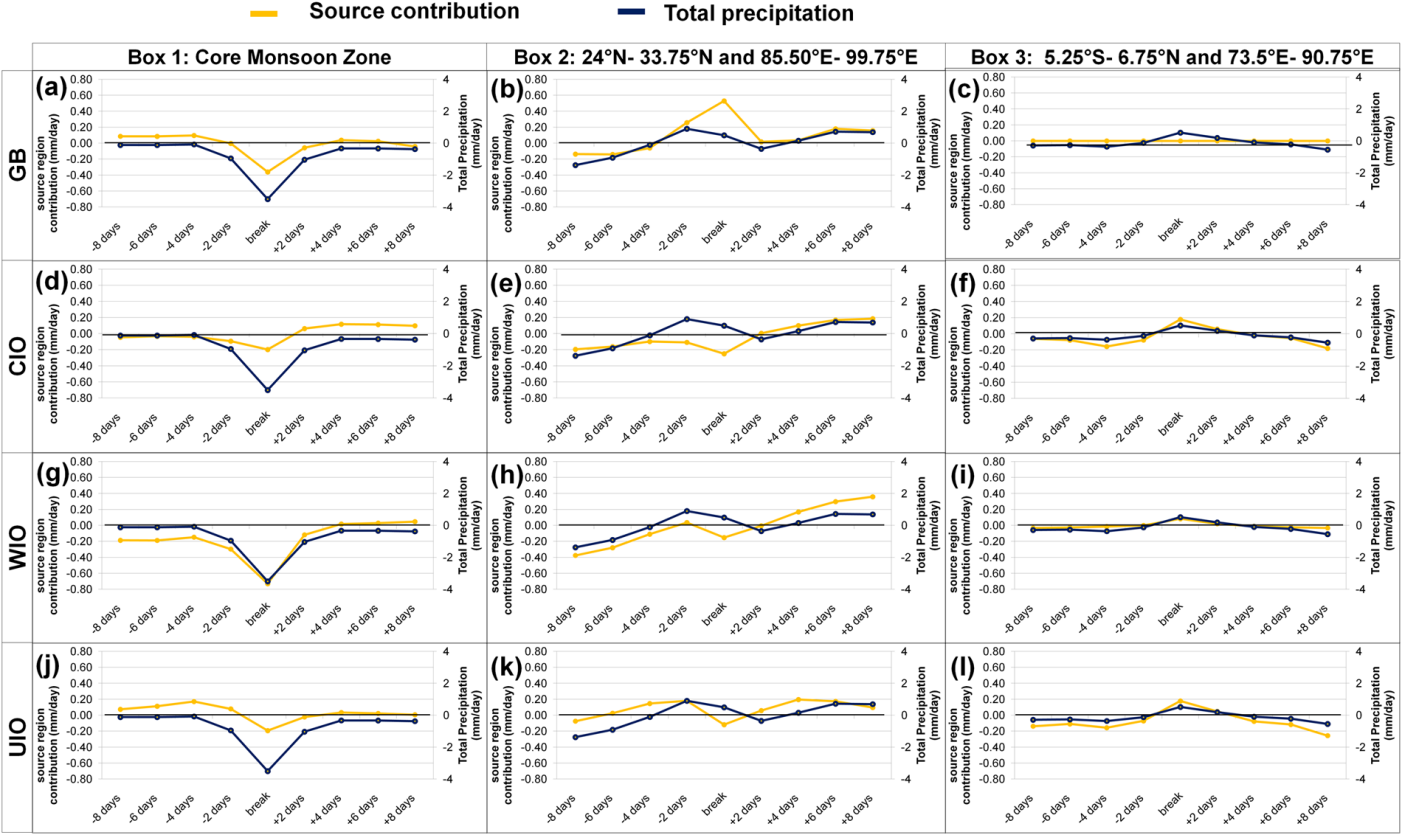
Same as Fig. 3 but for a break period. Plots are prepared using Microsoft Excel 2007 (https://products.office.com/en-IN/download-office-2007).

We find that the east–west rainfall dipole arises primarily due to the anomalous terrestrial moisture contribution to the CMZ and northeast India during active and break periods, respectively. The evolution of east–west asymmetry over the Indian region is explained by comparing the precipitation anomaly (the deviation from seasonal mean) across three selected regions [*1*, 2, and 3 as shown in Fig. 3(c)] along with the terrestrial moisture contribution from the GB during active [Fig. 3(a,b)] and break periods [Fig. 4(a,b)]. The precipitation anomaly and the moisture contribution from GB are observed to be increasing over core monsoon zone [*region 1* in Fig. 3(c)] prior to the active period; reach a maximum during the active period, and then decrease [Fig. 3(a)]. In contrast, both the precipitation anomaly and the anomaly of the moisture contribution from GB over *region 2* [Fig. 3(c)] show an opposite pattern [Fig. 3(b)], when compared with the pattern over *region 1*. A nearly opposite pattern is observed during break periods for precipitation anomalies and GB contribution over the CMZ [Fig. 4 (a)]. A decreasing moisture contribution from GB to the Indo–Gangetic Plain towards the break period is due to strong westward advection of moisture from GB to northeast India. Although the precipitation anomaly over CMZ shows an almost opposite sign, its pattern over *region 2* is slightly different [Fig. 4(b)]. Evolution of moisture contributions from GB to the precipitation during 8 days prior to active periods to 8 days after active periods are presented in Supplementary Fig. S4(a)–(g). Similarly, for break periods, they are presented in Supplementary Fig. S5(a)–(g). A maxima in moisture contribution from GB to *region 2* is observed during break periods (Supplementary Figures S4, S5, and S6), when the westerlies from northwest India replace the easterlies across CMZ and northeast India [Fig. 1(b)] and drive the moisture towards northeast India. The contributions from the GB to the NE India builds-up continuously starting from 2 days before breaks, reaches its peak during the break period when the GB-ET is maximum and then decrease afterwards (Supplementary Figure S6). The maximum ET values over the GB during the break period do not contribute to the recycling and precipitation over the CMZ, instead it contributes to the precipitation over NE India. Hence, a significant portion of the precipitation over the NE region is originated from the moisture accumulated from GB evaporation (nearly 2–3 days prior) in addition to evaporation from the other sources that falls along the trajectory. Similarly, the recycling values over the GB start to increase few days (~2 days) before the active periods, with the maximum recycling observed during the active periods (Supplementary Figure S6). The effect of precipitation recycling over GB extends even after the end of the active periods in a gradually decreasing manner for approximately 8 days. Contrary to that we observe a lower amount of precipitation recycling during break period over the GB due to increased advection of moisture from GB to the NE India [Fig. 1(b)].

**Figure 5:**
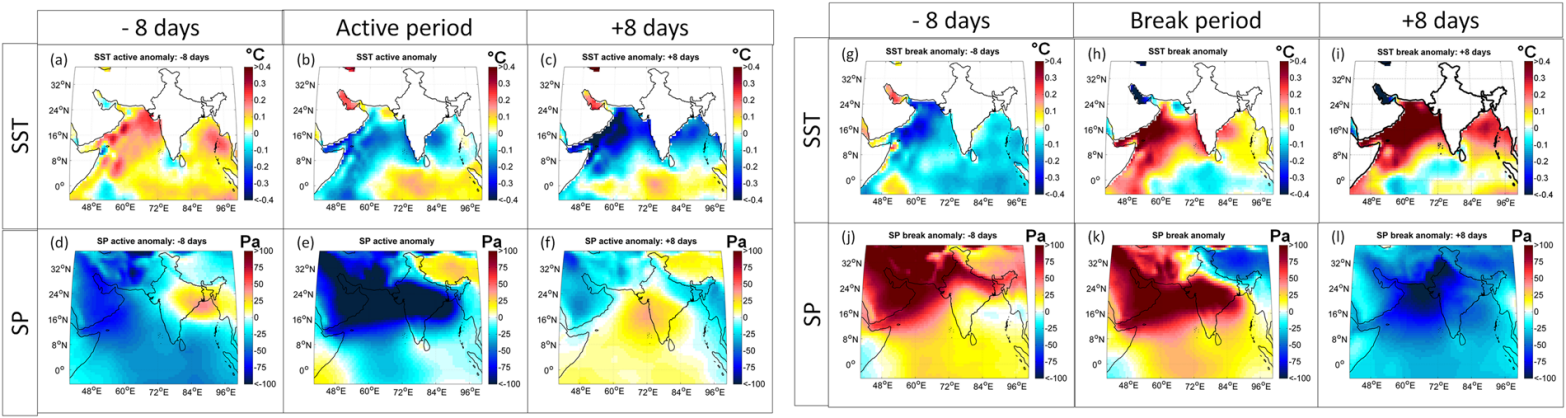
Sea Surface Temperature (SST)and surface pressure (SP) anomalies before, during, and after an active (left) and a break (right) period. The SST units are in *°C* and surface pressure are in *Pa*. The anomaly values are statistically significant at 0.05 level. 2-tailed student’s t-test is used for the same. Maps are prepared using MATLAB R2012b (http://in.mathworks.com/products/newproducts/release2012b.html). The terrestrial boundaries used in the plot are developed from the free spatial data provided by DIVA-GIS website (http://www.diva-gis.org/) and MATLAB R2012b.

It is also important to note that the terrestrial moisture contribution from GB is only associated with the oscillatory rainfall pattern between *region 1* and *region 2*. GB does not have a significant influence on precipitation over peninsular India (Supplementary Figures S4 and S5). Enhancement of GB contribution to *region 2* during breaks (Supplementary Figure S5) with the enhancement of strong subtropical westerlies^11^ can also be viewed as eastward migration of the monsoon trough due to the formation of the Iranian High^11^ and a weakening of the Tibetan High (Supplementary Figure S5) with its eastward shift. In contrast, there exists a strong Tibetan High with low pressure over CMZ during active periods [Fig. 5(e)]. Furthermore, strong easterlies [Fig. 1(a)] along the Indo-Gangetic Plain promote moisture recycling (although the evaporation is less) within the GB; a large moisture flux from the Bay of Bengal (part of UIO) also contributes to the precipitation over CMZ during active periods (Supplementary Figure S4).

### North–South pattern of rainfall during Active and Break period

A north–south dipole in rainfall anomaly during active/break periods arises primarily due to the fluctuations of northward propagating TCZ between the CMZ and the equatorial Indian Ocean^9^. A competition between these two favorable locations for the TCZ^1^ produces a north–south structure of rainfall anomaly^28^. The fluctuations of TCZ within the monsoon season and its impact on monsoon intraseasonal variability appear to be guided by the moisture transport from different oceanic sources to the Indian subcontinent.

Strengthening of continental TCZ over the Indian region is accompanied by westward propagating synoptic features from western Pacific through the Bay of Bengal and northward propagating synoptic systems from the equatorial Indian ocean^1^. The moist convection^1^ over the CMZ during active periods (Supplementary Fig. S6) is associated with the weakening of TCZ over the equatorial Indian Ocean [Supplementary Fig. S2(a)]. Here, to investigate this further, we compare the composites of anomalies of moisture contributions from different evaporative sources to the Indian monsoon region during active and break periods. A strong southwesterly moisture flux from WIO and easterly moisture flux from UIO is observed during the active period [Fig. 1(a)], accompanied by a strong easterly circulation from the Bay of Bengal and northeast India. Similarly, anomalies of circulation in opposite directions are observed during the break period [Fig. 1(b)]. WIO, which is one of the major sources of atmospheric moisture to ISMR^37^, has a positive anomaly of moisture contribution over entire India and Bay of Bengal during an active period [Fig. 2(c)]. In contrast, during a break periods, the WIO has a negative moisture contribution over the same region [Fig. 2(d)]. Central Indian Ocean also contributes positively to entire India during an active period [Fig. 2(e)] and negatively during a break period [Fig. 2(f)], although during the active period its contribution is maximum over the Bay of Bengal. It has been reported^28^ that some of the strong MISO events, initiated over the Indian Ocean become weak after reaching the southern peninsular India. Here, we find that the moisture contribution from CIO weakens after reaching peninsular India, whereas WIO contributes significantly to the precipitation over CMZ resulting in high positive precipitation anomaly during the active periods.

Upper Indian Ocean (UIO) through the Bay of Bengal contributes more during the active periods [Fig. 2(g)] and less during the break periods [Fig. 2(h)]. Interestingly, the Bay of Bengal appears to be less influential to the seasonal rainfall^37^ over the Indian subcontinent but has greater role during active spells over the CMZ. The formation of monsoon trough over the Indo-Gangetic Plain and the subsequent increase in convection result in larger amount of moisture transport from the UIO and GB to the CMZ.

Figures 3 and 4 show spatially averaged (averaging *region 1, region 2* and *region 3*) anomaly of precipitation and contributions from different moisture sources during active and break periods, respectively. Here, it is important to note that the north–south pattern in rainfall anomaly [Fig. 1(a,b)] arises primarily due to anomalous oceanic moisture supply. Oceanic sources (CIO, WIO, and UIO) have a strong north–south anomaly pattern over *region 1* and *region 3*, whereas the terrestrial source (GB) does not contribute to this north–south pattern [Fig. 3, and Fig. 4]. The moisture contribution from the oceanic sources to the CMZ are observed to be increasing prior to an active period, and decrease afterwards (Supplementary Figure S4), whereas opposite pattern is observed during a break period (Supplementary Figure S5). The contribution from WIO clearly shows a positive anomaly over the CMZ and a negative anomaly over the equatorial Indian Ocean [Fig. 2 (c,d,e,f)]. The contribution from the WIO to the CMZ is larger during active periods and smaller during break periods. Positive moisture anomalies from CIO and UIO to the CMZ (Fig. 2) can be seen as the weakening of TCZ over the equatorial Indian Ocean during CMZ active period and a reverse pattern is observed during break periods.

The SSTs over Arabian Sea and Bay of Bengal are relatively warmer prior to an active spell but are observed to be cooler during peak active periods^43^ [Fig. 5]. This is most likely due to the excess evaporative cooling associated with stronger winds over the Arabian Sea and convection over the Bay. This suggests that the warmer temperature prior to active events bring in more moisture from the Indian Ocean to the Indian subcontinent. Our results are consistent with findings of Roxy and Tanimoto(2007)^44^, which highlights the fact that the positive SST anomalies in the Arabian Sea, prior to an active period, tend to form a favorable condition for convective activity and enhanced precipitation over peninsular India. These move further northeastward and merge with the northward propagating precipitation anomalies from the Bay of Bengal, enhancing the conditions for active period over the core monsoon region also. Their study also shows that the underlying positive SST anomalies enhance the northward propagating precipitation anomalies over the Arabian Sea thermodynamically. An opposite patterns of SST and resulting moisture transport are observed during a break period. The patterns of surface pressure anomalies as observed during active and break periods (Fig. 5) are more consistent with the transport of moisture from WIO than any other sources and clearly, this is the most important source contributing to the intra-seasonal variations in ISM. Recent studies^45,46^ have found significant warming over the WIO as compared to other parts of the Indian Ocean and its impacts are clearly evident with the changing characteristics of intraseasonal variations^25^ over recent decades.

### Propagations of Intraseasonal Variability (ISV) through Moisture Source Contributions

Intraseasonal variations operate at multiple timescales. Hence an accurate modeling of these features is of utmost importance in terms of agricultural productivity and socio-economic relevance. Therefore, in this context the role of individual moisture sources in the northward and westward propagation must be quantified for a realistic simulation and prediction of the ISM by the numerical models. It is reported in earlier studies^9,17,21,22,28,47^ that the northward and eastward propagations of intraseasonal variations of summer monsoon rainfall are prominently observed in a low frequency (30–60 day) signal; while the westward propagation is generally observed in the high frequency (10–25 day) signal. Here, to explore this further, we have performed a detailed lead/lag regression analysis of the moisture contributions from different sources with 10–25 days and 30–90 days band pass filters (details in methods).

**Figure 6:**
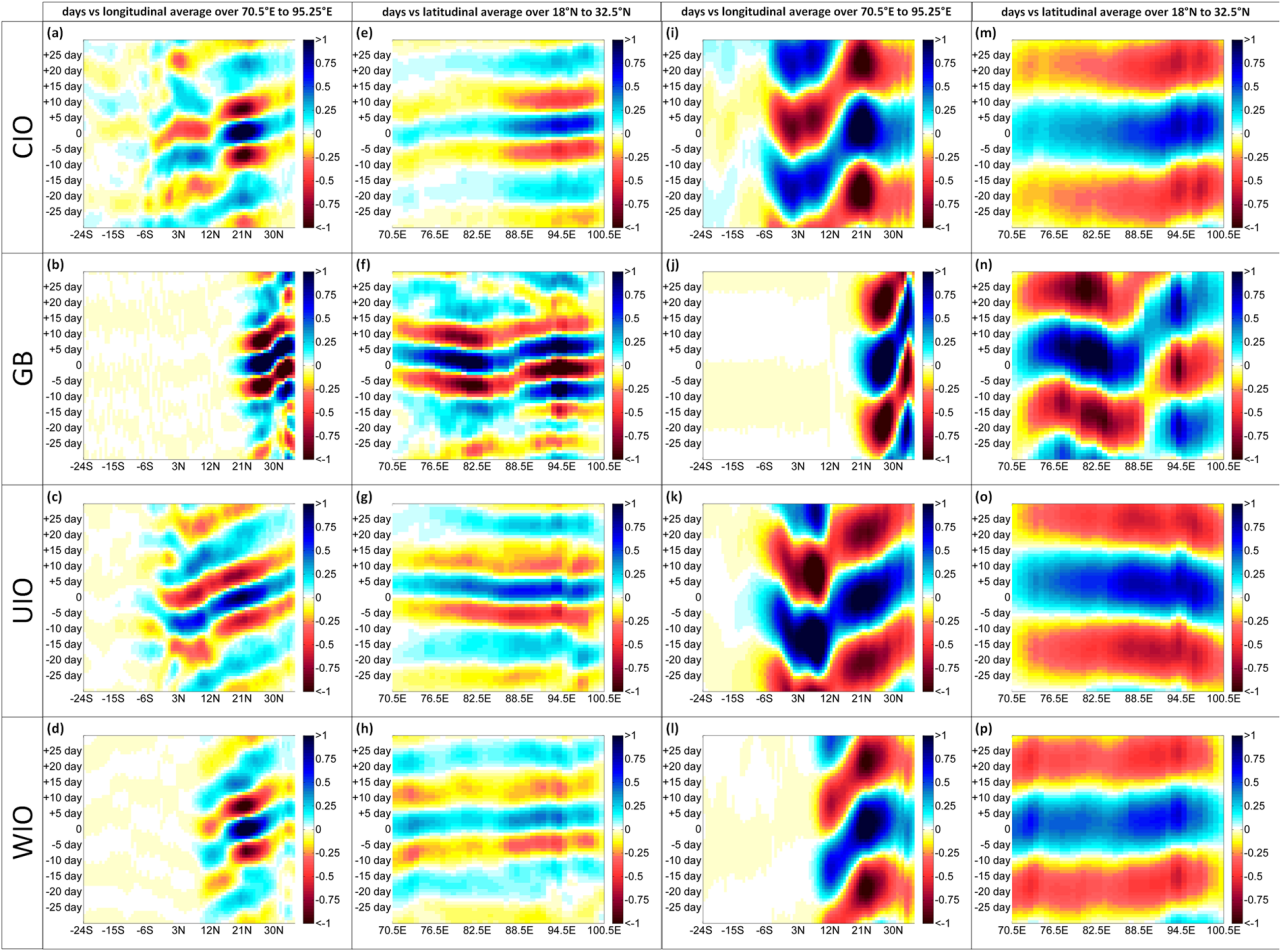
Hovmöller plots of regressed 10–25 day and 30–90 day filtered precipitation anomalies (mm/day) averaged over 70–90°E (left) and averaged over 18–32.5°N (right) with respect to a reference time series area averaged over Indian region (12–22°N and 70–90°E). Maps are prepared using MATLAB R2012b (http://in.mathworks.com/products/newproducts/release2012b.html).

We observe that the northward propagations at 30–90 day frequency ranges are clearly visible in the anomalies of moisture contributions from the three oceanic sources, WIO, CIO, and UIO (Fig. 6). However, the anomalies of contributions from the oceanic sources have very small (negligible) impact on eastward and westward propagations of MISO. The contribution from the terrestrial source GB shows both eastward and westward propagation at the 30–60 day frequency range. Furthermore, a strong high frequency (10–25 days) eastward and westward propagation of intraseasonal variability is observed in the contribution anomaly from the terrestrial source GB. The anomalies of contribution from the oceanic sources such as CIO and WIO also show a slight eastward propagation; in addition to that their impact can also be seen in the high frequency northward propagations. The detailed results are tabulated in Supplementary Table S1.

Supplementary Figure S7 shows the space-time evolution of MISO and its relation to the contributions from the three oceanic sources (CIO, UIO,WIO). The initiated (day −15) convection from the equatorial Indian Ocean propagates northward towards the Indian subcontinent and on subsequent days (day +5) it also moves northwestward across the central region of the India. The northward propagating CIO anomalies enter the subcontinent from the Arabian Sea direction, whereas UIO anomalies enter the subcontinent through the Bay of Bengal and then extend northwestward across the CMZ. The anomalies of the WIO contribution, originate from an oceanic source north of the equator and propagate northwards to the Indian region. It is important to note here that the timescale and spatial variations of these three contributions (CIO, UIO, and WIO) are notably different from each other. We also observe a slower rate of phase change in UIO anomalies. The variations in the time scales of propagations of the contributions from different sources likely due to of the distinct air-sea interaction processes over the UIO compared to the WIO and CIO^48,49^. However, this needs to be analyzed through model driven hypothesis testing and may be considered as a future area of research.

We observe that both the terrestrial sources and oceanic sources, especially the WIO and GB, have a strong association with the amplitude of the precipitation anomaly over the Indian subcontinent (Fig. 2). The moisture transport from WIO during active periods contributes strongly to the precipitation over the CMZ, northeast India, and Bay of Bengal and hence, it contributes significantly to the mean field. GB contributes to the CMZ during active periods and to the northeast India during break periods with negligible moisture transport to peninsular India. Hence, it contributes mostly to the east-west anomaly and least to the north-south anomaly. Furthermore, among the oceanic sources WIO contributes significantly to mean precipitation anomaly over the CMZ, whereas the contributions from the CIO and UIO to the precipitation over CMZ is relatively small. We also observe that the CIO during active periods contributes more to the Western Ghats and Bay of Bengal. It should be noted that the anomaly of moisture contributions from WIO to the northeast India is positive during active periods; however the negative anomaly of contributions from GB to the same sink region dominates, resulting in a mean negative anomaly of precipitation during break periods with an east-west pattern. Here, it is important to note that the distinctive pattern of mean precipitation anomaly observed in the Fig. 1(a,b) represents the mean effect of moisture contribution from all the sources. Oceans contribute to the north-south anomaly pattern whereas terrestrial source such as GB contributes to the east-west asymmetry of precipitation anomaly.

## Conclusions

The present study shows that the east–west and north–south dipoles in intraseasonal rainfall anomalies over India during active and break periods are governed by the moisture transport from oceanic and terrestrial evaporative sources to the rainfall during the Indian summer monsoon. The key findings of the present work are:

- Among the oceanic sources, the intraseasonal variability of summer monsoon rainfall is mainly governed by the moisture contributions from the WIO, while among the terrestrial sources influence of GB is dominant.
- The north–south feature during active and break periods is due to the large–scale oceanic moisture transport to the core monsoon zone; whereas the east–west rainfall anomaly pattern is influenced by both local terrestrial component and large–scale westerlies.
- The lead/lag regression analysis of contributions from different moisture sources with 10–25 days and 30–90 day band pass filters reveals that the three oceanic sources (WIO, CIO, and UIO) are associated with northward propagating 30–60-day oscillation of monsoon ISVs whereas, the terrestrial source GB is related to eastward and westward propagating 10–20-day oscillation of monsoon ISV.
- The westerly jet over the Indo–Gangetic Plain plays an important role in monsoonal activities over the core monsoon zone. Increased westerlies during a break period replace the easterlies from the Bay of Bengal and northeast India and also reduce the recycling within the GB.
- The formation of the monsoon trough along the Indo–Gangetic Plain and the increased convection demand a large moisture transport from the UIO and GB into the core monsoon zone. Additionally, a prominent southwesterly jet from the WIO and CIO brings a large amount of moisture to India during active spells. Recycling within the Ganga Basin is also observed to be prominent during active periods.

We posit that this methodology and our results offer a stringent test for the models in terms of their rendition of the coupled Indian monsoon and hence to the entire monsoon system which is critical considering the deficiencies that have been reported in monsoon predictions and projections^50–52^.

## Data used and Methods

### Data used

In the present study we use the global ERA–Interim^40^ (European Centre for Medium–Range Weather Forecasts Reanalysis) reanalysis dataset, available at 0.75° spatial resolution. We use the vertical integral of eastward and northward water vapor flux *(Q*_*x*_, and *Q*_*y*_) to obtain the moisture weighted–wind component in two horizontal directions (*U* = *Q*_*x*_ */W*, *and V* = *Q*_*y*_ */W)*. The atmospheric pressure level data used for this study are from 1000 hPa to 300 hPa. We also use daily average of evaporation *(E)*, total column water *(W)*, and precipitation *(P)*, for 1979–2013. Additionally, variables such as Sea Surface Temperature *(SST)*, Geopotential Height at 500 mb, Outgoing Long wave radiation and Surface Pressure are also used to understand various physical processes during active and break periods.

### Identifications of Active and Break periods

Active and break periods during the Indian summer monsoon are extensively studied in recent decades; different approaches have beenemployed^2,4,10,13,53,54^ to identify active and break periods. Here, we invoke the most recent method^15^ to identify active and break spells which is based on standardized rainfall anomaly over the core monsoon zone. The monsoon rainfall over the CMZ is significantly correlated^15^ with the rainfall over major parts of India, except for the Himalayan foothills, and northeast India. Standardized rainfall anomalies are calculated by averaging the daily (5–day moving average) rainfall over the CMZ and standardizing the rainfall time series by subtracting its long term climatology, and dividing with its daily standard deviations. Active periods are defined as the periods during which standardized rainfall anomalies are greater than +1.0 for at least three consecutive days. Similarly, break periods are defined as the periods during which standardized rainfall anomalies are less than −1.0 for at least three consecutive days. The time period considered^53^ for identification of active and break periods is between the dates of 15th of June and 15th September for the years 1980 to 2014.

### Quantification of precipitation contribution from different evaporative sources

In the present study, we have subdivided the identified monsoon domain (30°E to 135°E and 55.5°N to 40.5°S) into 20 terrestrial and 7 oceanic regions (Supplementary figure S3). Here, the terrestrial region boundaries are approximately selected on the basis of uniform climate subtype^55^ and percent forest cover^56^. The oceanic sources are subdivided into 7 different regions based on moisture flux convergence using vertically integrated divergence of moisture flux obtained from ERA–Interim (European Centre for Medium–Range Weather Forecasts Reanalysis) reanalysis dataset. In the present study we have used an extended version of dynamic recycling model^37,38^ which is based on a Lagrangian approach with an assumption of a well-mixed atmosphere, to quantify the impact of atmospheric transport of water vapor from different evaporative sources, on the intraseasonal variability of Indian summer monsoon rainfall.

For a domain *D* which consists of *N* different evaporative sources, the fraction of moisture collected from *N* different source regions 1, 2, *3*, ……, *N*, is represented by *R*_1_, *R*_2_, *R*_3_, ………, *R*_*N*_, respectively. A detailed description of the moisture fraction calculation is provided in Dominguez *et al*.^39^, and Martinez and Dominguez (2014)^38^. The total contribution at sink $(\chi ,\xi ,t)$ from all the segments within the source region ‘*SR*_*k*_’ and along the trajectory of the water vapor are grouped as

1 $${\alpha }_{S{R}_{k}}(\chi ,\xi ,t)=\sum {S}_{i}\in S{R}_{k}({\prod }_{j=1}^{{S}_{i}-1}{\alpha }_{j}(\chi ,\xi ,t)){R}_{{S}_{i}}(\chi ,\xi ,t)$$

Here, *Si* represents the *i* ^*th*^ segment. ‘*α*_*j*_’ represents the fraction of evaporated moisture from region ‘*SR*_*k*_’, which is not lost (via precipitation) in the intermediate part of the trajectory to the sink region. Therefore, net moisture contributions from a source ‘*SR*_*k*_’ to the precipitation over a sink $(\chi ,\xi ,t)$ can be calculated by multiplying the moisture fraction (equation 1) corresponding to the source region *‘SR*_*k*_’ with a precipitation $P(\chi ,\xi ,t)$ at that sink location for day *‘t’*,

2 $$P{R}_{S{R}_{k}}(\chi ,\xi ,t)={\alpha }_{S{R}_{k}}(\chi ,\xi ,t)\,\ast \,P(\chi ,\xi ,t).$$

Similarly, the total contributions from all the segments, including those that are within the source region along the trajectory can be calculated by adding contribution from all segments of the trajectory. The spatially averaged contribution in terms of precipitation *P*_*r*_ from source region *SR*_1_ to the sink region *SN*_1_, for any day *t* = *d* can be calculated by

3 $${P}_{r}(S{R}_{1},S{N}_{1},t)=\frac{{\sum }_{(\chi ,\xi )\in S{N}_{1}}{\alpha }_{S{R}_{1}}(\chi ,\xi ,t)P(\chi ,\xi ,t)\delta A(\chi ,\xi )}{{\sum }_{(\chi ,\xi )\in S{N}_{1}}\delta A(\chi ,\xi )},$$

and,

4 $${R}_{r}(S{R}_{1},S{N}_{1},t)=\frac{{\sum }_{(\chi ,\xi )\in S{N}_{1}}{\alpha }_{S{R}_{1}}(\chi ,\xi ,t)\,\ast \,P(\chi ,\xi ,t)\,\ast \,\delta A(\chi ,\xi )\,\ast \,100}{{\sum }_{(\chi ,\xi )\in S{N}_{1}}P(\chi ,\xi ,t)\delta A(\chi ,\xi )},$$

where ${P}_{r}(S{R}_{1},S{N}_{1},t)$ and ${R}_{r}(S{R}_{1},S{N}_{1},t)$ represents the precipitation generated and percentage precipitation over ‘*SN*_1_’ as a result of evaporation ‘*SR*_1_’. Here, $\delta A(\chi ,\xi )$ represents the area of each grid cell.

Supplementary Figure S3 shows the seasonal precipitation contribution from different evaporative sources, highlighting the major sources (Supplementary figure S3(b)) contributing to All–India Monsoon Rainfall (using equation 4). We analyzed the role of atmospheric moisture transport in monsoon intraseasonal variability due to each of the major evaporative sources. The anomaly values, which are statistically significant at 0.05 level, are considered for the analysis and figures.

### Calculation of band pass filtered daily anomalies

We study the regressed 30–90 day and 10–25 day band pass filtered daily anomalies of precipitation which is computed by following the procedure similar to Sabeerali *et al*.^26^. The daily anomaly is calculated by subtracting daily smoothed (mean + 1st three harmonics) long term climatology from the dataset. A filtered JJAS rainfall anomaly averaged over12°N–22°N, 70°E-90°E, is used as a reference time series for regression. Similarly the regressed filtered anomalies of moisture contributions from four major sources the WIO, CIO, GB, and UIO are computed. The composite evolution of the ISOs across the filtered anomalies of four variables (Precipitation, WIO, CIO, and UIO contributions) is captured by computing the composites at different lags. Then the results are presented in the form of Lag-longitude (averaged over 18°N to 32.5°N) and Lag-Latitude (averaged over 70.5°E to 95.25°E) filtered precipitation and contributions anomalies.

### Data Availability

All the data used in the analysis are available open source (Mentioned in the text). The rainfall data is available from India Meteorological Department.

## Electronic supplementary material

Supplementary Information
