# Supplementary material for: Role of Oceanic and Terrestrial Atmospheric Moisture Sources in Intraseasonal Variability of Indian Summer Monsoon Rainfall
Source: Sci Rep. 2017 Oct 6;7:12729. doi: 10.1038/s41598-017-13115-7 (PMC5630610; doi:10.1038/s41598-017-13115-7)
Supplement: Supplementary file 1 — Supplementary Information [file 41598_2017_13115_MOESM1_ESM.pdf]

Supplementary Information for:

Role of Oceanic and Terrestrial Atmospheric Moisture Sources in Intraseasonal Variability of Indian Summer Monsoon Rainfall

Amey Pathak[1], Subimal Ghosh[1,2*], Praveen Kumar[3], Raghu Murtugudde[4]

[1]Department of Civil Engineering, Indian Institute of Technology Bombay,

Mumbai – 400 076, India.

[2]Interdisciplinary Program in Climate Studies, Indian Institute of Technology

Bombay,

Mumbai – 400 076, India.

[3]Civil and Environmental Engineering, University of Illinois at Urbana–

Champaign,

2527B VenTe Chow Hydrosystems Laboratory, 301 N. Mathews Ave., Urbana,

IL 61801

[4]Earth System Science Interdisciplinary Center (ESSIC)/DOAS, University of

Maryland, College Park, Maryland, USA

[*]Corresponding Author: subimal@civil.iitb.ac.in

Supplementary Table S1: Comparison of regressed 30-90 day and 10-25 day filtered anomalies of different moisture sources WIO, CIO, GB, and UIO.

Sr. No.	Source	30-90 day filtered anomaly	10-25 day filtered anomaly
1.	WIO	northward	northward and slightly eastward
2.	CIO	northward	northward and eastward
3.	GB	westward and eastward	westward and eastward
4.	UIO	northward and slightly westward	northward and slightly westward

Supplementary Figures:

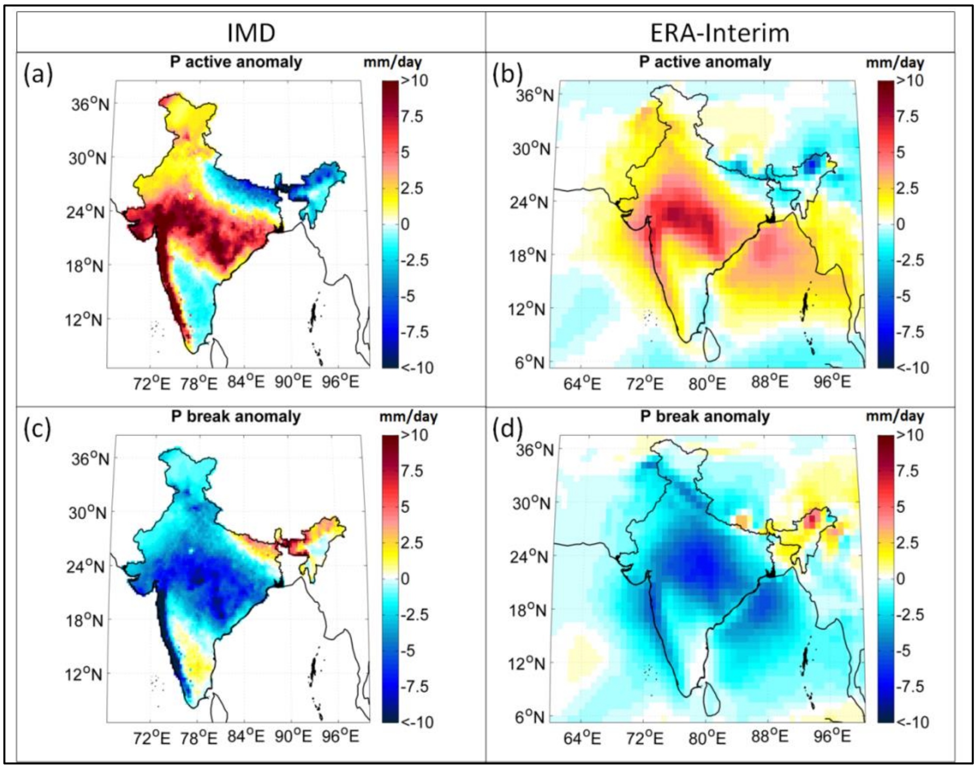

S1: Precipitation anomaly during Active (a, b) and Break (c, d), observed in IMD and ERA-Interim data respectively). The color scale runs from <-10 to >10 in intervals of 2.5 *mm day*$^{-1}$. The anomaly values are statistically significant at 0.05 level. Maps are

prepared using MATLAB R2012b (http://in.mathworks.com /products/newproducts/release 2012b.html). The terrestrial boundaries used in the plot are developed from the free spatial data provided by DIVA-GIS website (http://www.diva-gis.org/) and MATLAB R2012b.

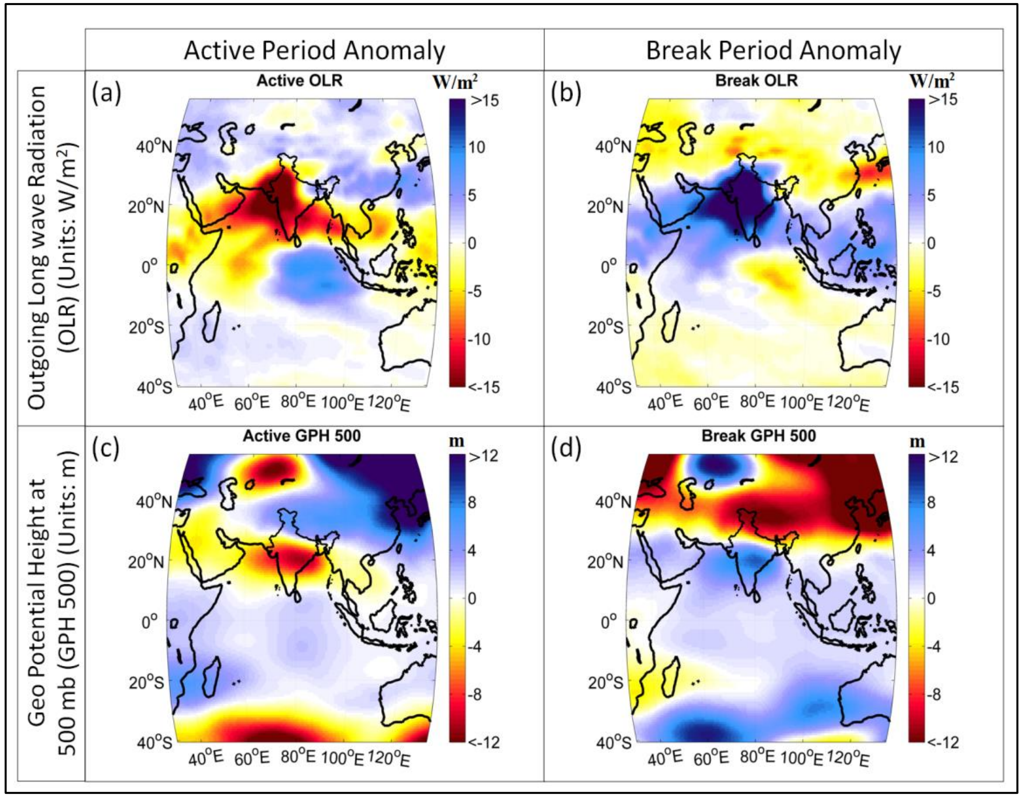

S2: Outgoing Longwave Radiation (OLR) anomaly and geo–potential height anomaly at 500 mb during Active (a, c) and Break (b, d), respectively. The anomaly values are statistically significant at 0.05 level. Maps are prepared using MATLAB R2012b (http://in.mathworks.com/products/

newproducts/release2012b.html). The terrestrial boundaries used in the plot are developed from the free spatial data provided by DIVA-GIS website (http://www.diva-gis.org/) and MATLAB R2012b.

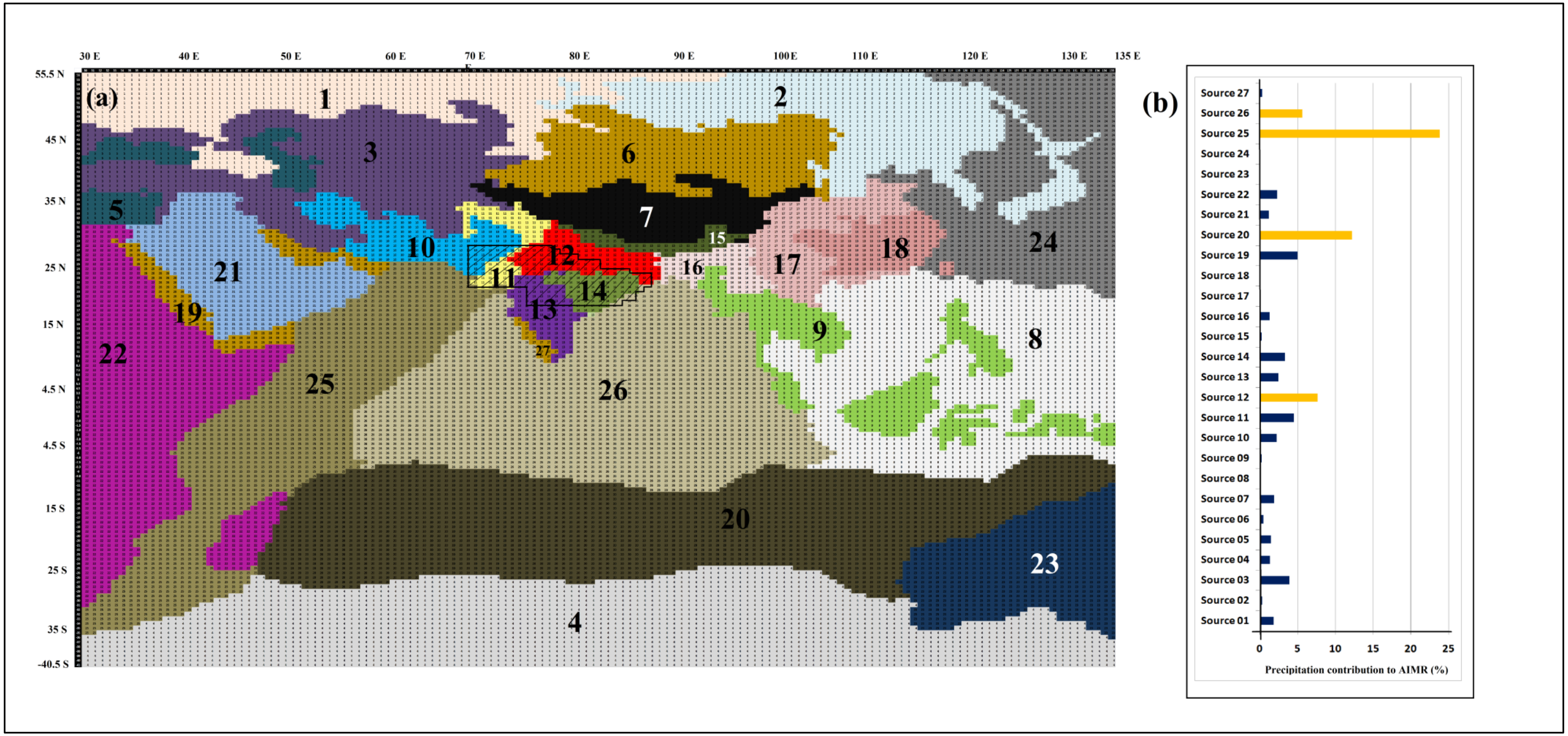

S3: Regions considered as the sources of moisture to ISMR (a). Regions over the ocean are based on vertically integrated moisture flux divergence, and land regions are defined by similar (approximately) climate subtype (Koppen climate classification: Kottek et al., 2006). (b) Ganga Basin

(GB) is observed to be main terrestrial source for All–India Monsoon Rainfall (AIMR), whereas among oceanic sources central Indian Ocean (CIO), Western Indian Ocean (WIO), and Upper Indian Ocean (UIO) contributes significantly to the AIMR. Maps are prepared using Microsoft Excel 2007 (https://products.office.com/en-IN/download-office-2007)

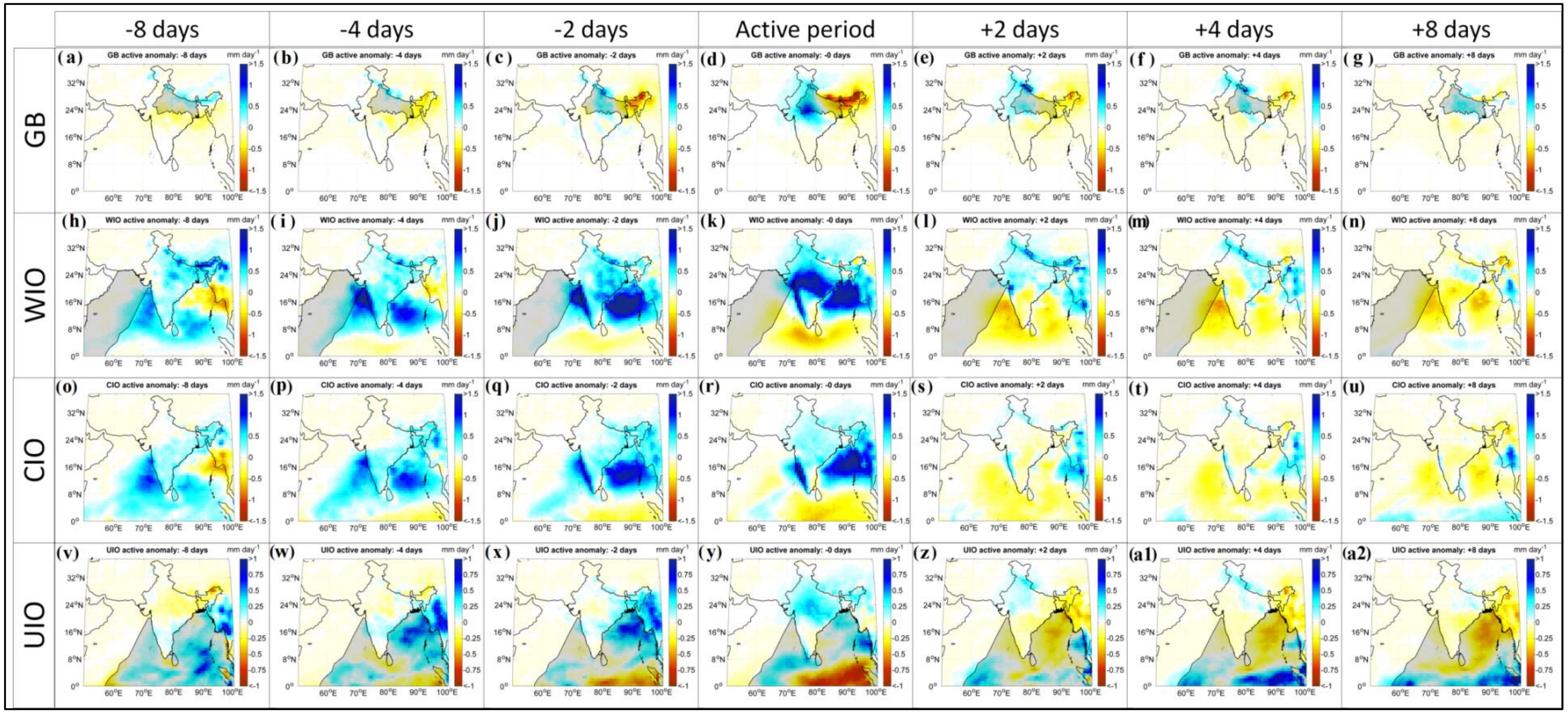

S4: Lag/lead day moisture contribution anomaly (units in *mm day*$^{-1}$) to precipitation from different evaporative sources viz. GB, WIO, CIO, and UIO, before-during-after an active period. The anomaly values are statistically significant at 0.05 level. Maps are prepared using MATLAB R2012b

(http://in.mathworks.com/products/new_products/ release2012b.html). The terrestrial boundaries used in the plot are developed from free spatial data provided by DIVA-GIS website (http://www.diva-gis.org/), Pathak et al.[37] and MATLAB R2012b.

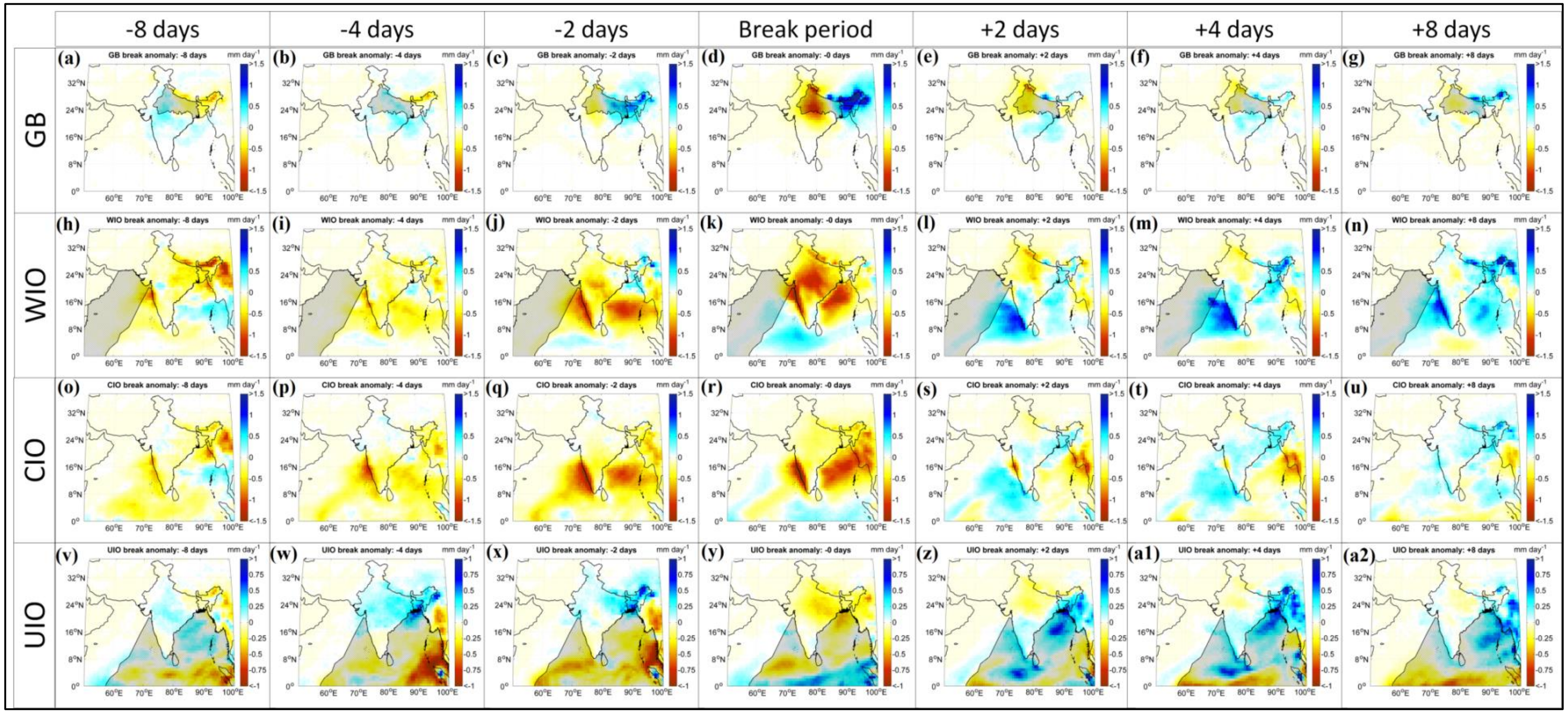

S5: Lag/lead day moisture contribution anomaly (units in *mm day*$^{-1}$) to precipitation from different evaporative sources viz. GB, WIO, CIO, and UIO, before, during, and after break period. The anomaly values are statistically significant at 0.05 level. Maps are prepared using MATLAB

R2012b (http://in.mathworks.com/products/new_products/ release2012b.html). The terrestrial boundaries used in the plot are developed from free spatial data provided by DIVA-GIS website (http://www. diva-gis.org/), Pathak et al.[37] and MATLAB R2012b.

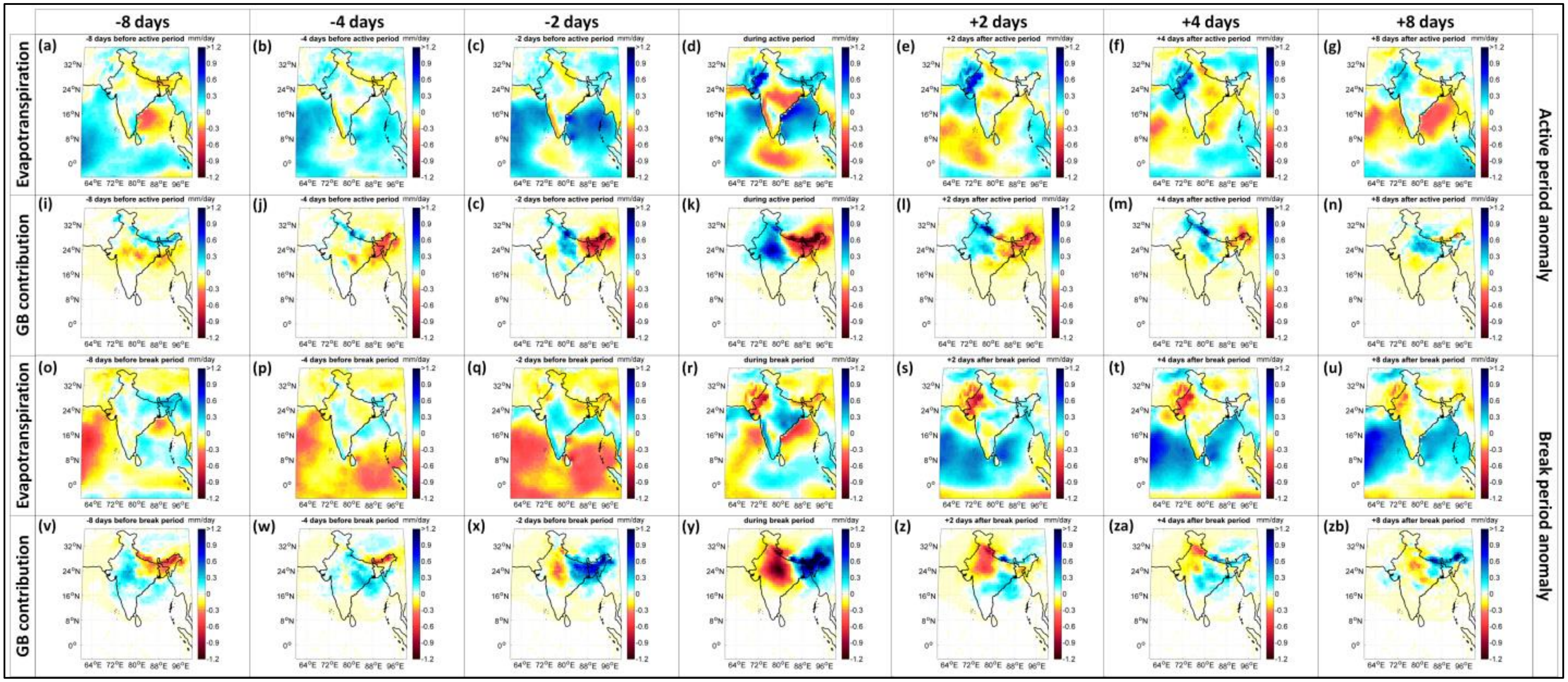

S6: The anomaly of moisture contribution from GB and the ET values before, during, and after active and break periods, respectively. Maps are prepared using MATLAB R2012b (http://in.mathworks.com/products/new_products/ release2012b.html). The terrestrial boundaries used in the plot are developed from free spatial data provided by DIVA-GIS website (http://www. diva-gis.org/) and MATLAB R2012b.

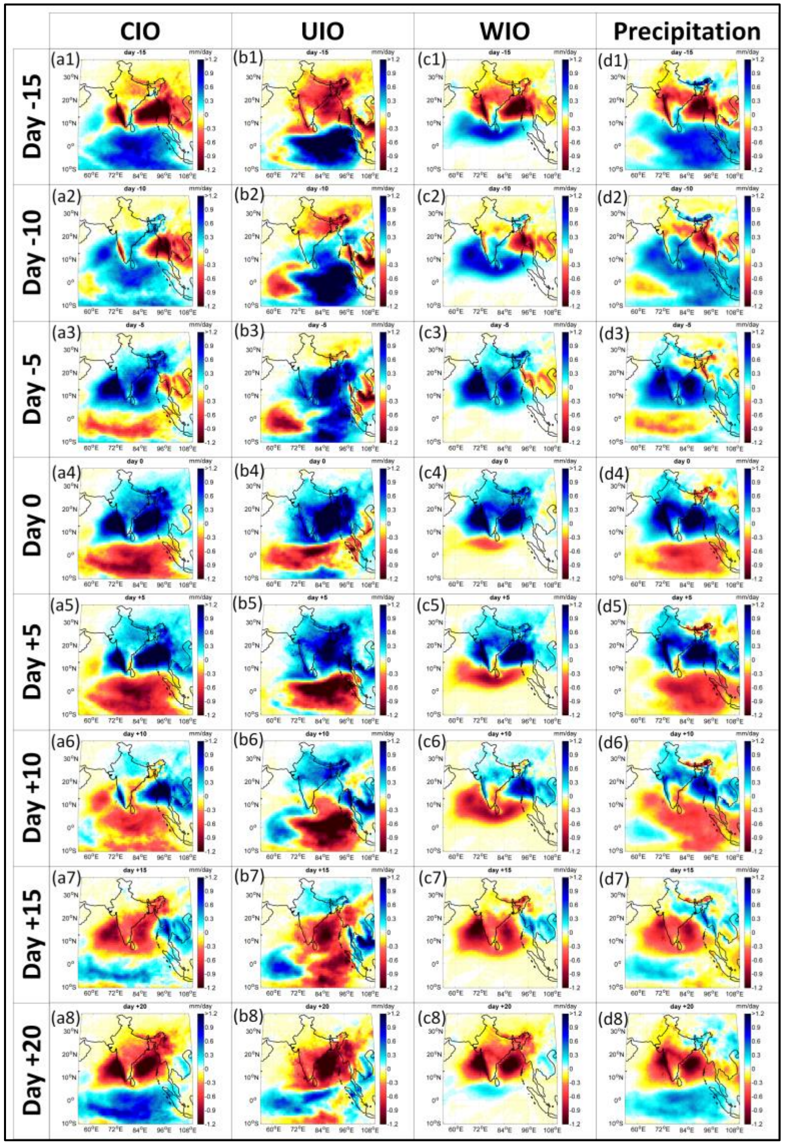

CIO
UIO
WIO
Precipitation
Day -15
Day -10
Day -5
Day 0
Day +5
Day +10
Day +15
Day +20
(a1)
(b1)
(c1)
(d1)
(a2)
(b2)
(c2)
(d2)
(a3)
(b3)
(c3)
(d3)
(a4)
(b4)
(c4)
(d4)
(a5)
(b5)
(c5)
(d5)
(a6)
(b6)
(c6)
(d6)
(a7)
(b7)
(c7)
(d7)
(a8)
(b8)
(c8)
(d8)

S7: Space-time evolution of the regressed 30 to 90 day band pass filtered precipitation anomalies (mm day^{-}1) with respect to a reference time series created by averaging the filtered precipitation anomalies over the central India region (12-22°N and 70–90°E). Maps are prepared using MATLAB R2012b (http://in.mathworks.com/products/new_products/ release2012b.html). The terrestrial boundaries used in the plot are developed from free spatial data provided by DIVA-GIS website (http://www. diva-gis.org/) and MATLAB R2012b.
